# Perioperative Glucagon-Like Peptide-1 Receptor Agonists (GLP-1RA) and Gastric Point Of Care Ultrasound (POCUS)

**DOI:** 10.24908/pocus.v9i2.17700

**Published:** 2024-11-15

**Authors:** Sivasenthil Arumugam, Hari Kalagara

**Affiliations:** 1 School of Medicine, University of Connecticut Farmington, CT USA; 2 Woodland Anesthesiology Associates Hartford, CT USA; 3 Mayo Clinic Jacksonville, FL USA

**Keywords:** POCUS, Gastric POCUS, Perioperative POCUS, Anesthesiology, GLP-1 Agonist

## Abstract

Glucagon-like peptide-1 receptor agonists (GLP-1 RA) are frequently used for diabetes and weight loss management. The GLP-1 RA   drugs delay gastric emptying and are a concern for increased risk of aspiration in the perioperative period. Current recommendations to hold these medications before surgery are consensus based. Gastric point of care ultrasound (POCUS)   can provide information regarding nature and volume of gastric contents in these patients during the perioperative period. In this case series, we present three patients where gastric POCUS helped formulate a safer, alternative anesthetic plan. Anesthetic management varied depending on the situation, urgency and needs of the procedure. We recommend gastric POCUS in this group of patients to provide objective assessment of gastric contents.

## Introduction

Glucagon-like peptide-1 receptor agonists (GLP-1 RA) are increasingly used for diabetes and weight loss management. Among the surgical patients taking GLP-1 RA, the recent focus has been on delayed gastric emptying that can increase the risk of airway aspiration. Current recommendations on the perioperative use of these drugs and fasting are consensus based due to lack of strong evidence. Gastric point of care ultrasound (POCUS) features prominently in these recommendations as an objective tool. Bedside ultrasound assessment of gastric contents can provide valuable information to the anesthesiologist for safe airway and anesthetic management.

We present three different cases where gastric POCUS played a crucial role in making a safe anesthetic plan. Anesthetic plans were altered or individualized depending on the surgical procedure, urgency of the operation, safe outcome and patient satisfaction.

## Case Reports

### Case #1

**Figure 1:**
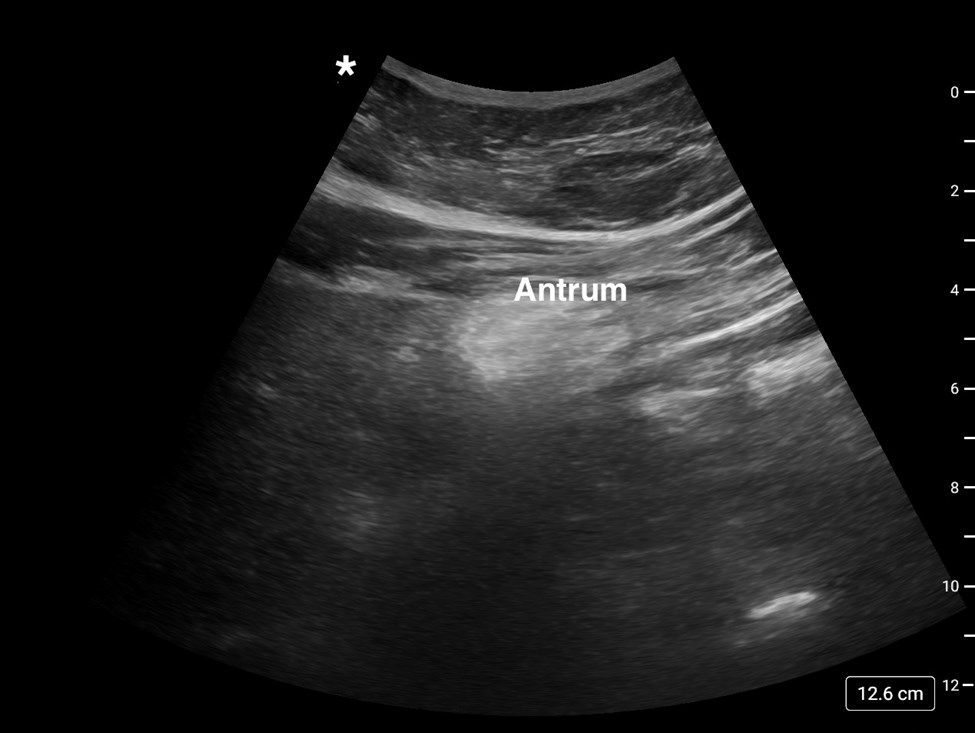
Gastric ultrasound image case #1

 A 68-year-old man taking Semaglutide (Ozempic^®^) for diabetes management presented for trigger finger surgery. He was scheduled for surgery about a week after his last dose of GLP-1 RA. Despite fasting overnight, gastric POCUS (Figure 1) performed on the morning of surgery revealed solid gastric contents (full stomach). After discussing with the patient and surgeon, the surgery was canceled as it was a completely elective procedure. The usual anesthetic plan for trigger finger repair would have been intravenous sedation with monitored anesthesia care (MAC). Solid gastric contents revealed by the gastric POCUS prompted a discussion with the patient and the surgeon. The surgery was canceled and rescheduled later with alteration in preoperative fasting duration with possible change in duration of stopping Semaglutide.

The patient was consulted to Endocrinology, scheduled three weeks later (21 days off GLP-1 RA) with only liquids for 24-hours before surgery. Gastric POCUS performed on the rescheduled day was not significant, and he had an uneventful surgery under MAC.

### Case #2

A 32-year-old woman presented for right shoulder arthroscopic labral and rotator cuff repair with biceps tenodesis. Medical history included exercise induced asthma, hypothyroidism, obesity and depression. She was also taking Tirzepatide (Mounjaro^®^) once a week for weight loss along with a few other medications for medical conditions. Her last dose of Tirzepatide was more than a week back.

**Figure 2:**
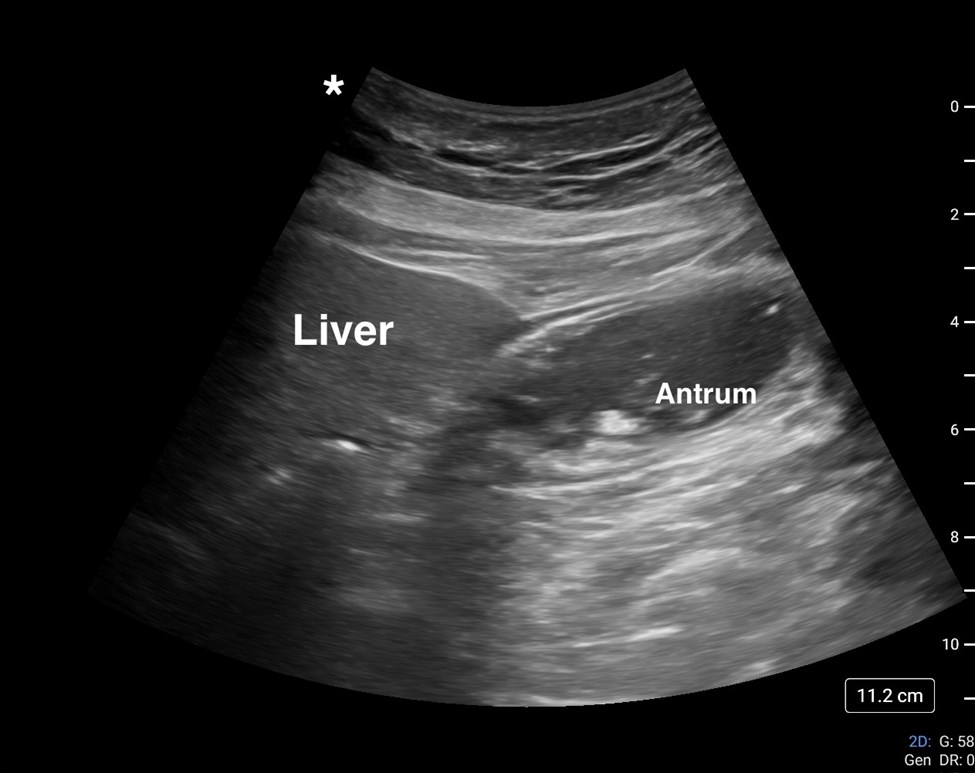
Gastric ultrasound image case #2

Gastric POCUS in preoperative assessment revealed particulate (late-stage solid) gastric contents (Figure 2). The patient and surgeon discussed the implications, risks, and options given the gastric POCUS findings. A decision was made to proceed with rapid sequence induction and intubation. A preoperative ultrasound guided right interscalene block was performed in the holding area before proceeding to the operating room. The surgery and post-operative phase were uneventful, and the patient was discharged home later that day.

Most shoulder arthroscopic surgeries are performed under general anesthesia (GA) with laryngeal mask airway (LMA), after placement of an interscalene block. The findings of the gastric POCUS exam guided our decision to avoid LMA and consider surgery only with a safely secured and protected airway.

### Case #3

**Figure 3:**
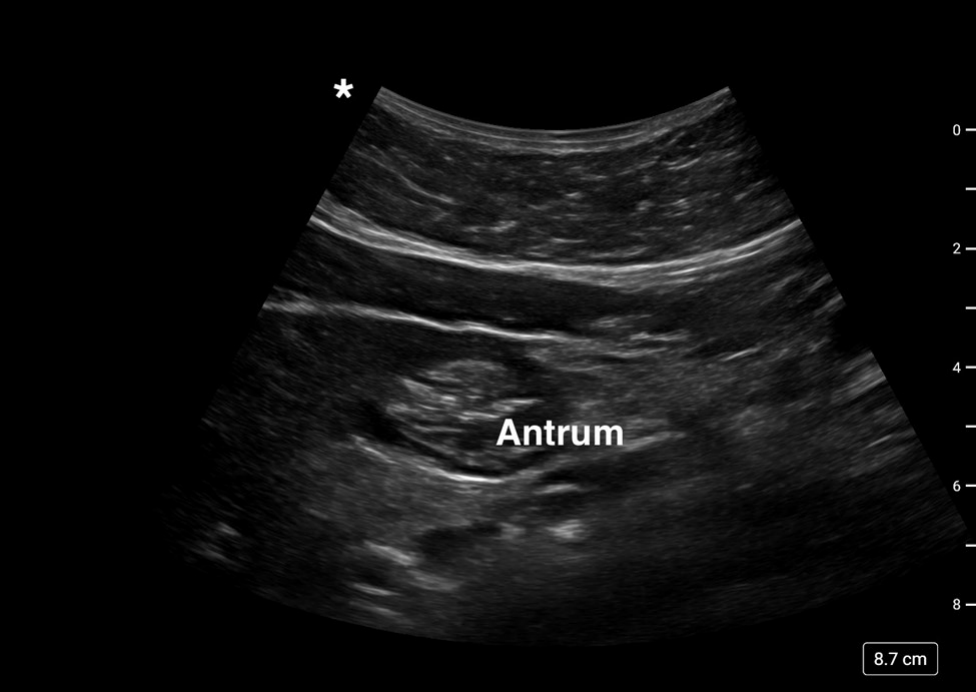
Gastric ultrasound image case #3

A 72-year-old man with a history of hypertension, high cholesterol, type-2 diabetes, asthma and arthritis presented for a same day total knee arthroplasty. He was on Semaglutide (Ozempic^®^) for diabetes and the last dose was eight days back. The patient had fasted overnight and had only liquid diet for 24 hours prior to surgery. Gastric POCUS on the morning of surgery showed significant gastric volume in both supine and right lateral decubitus (RLD) positions (Figure 3). A discussion with the patient and the surgeon regarding the volume of gastric content ensued and everyone agreed there would be spinal anesthesia with very minimal sedation for the operative procedure.

An ultrasound guided adductor canal block and iPACK (Infiltration between popliteal artery and capsule of knee) were performed in the preoperative holding area. In the operating room, spinal anesthesia was performed with the patient in a sitting position. The patient was positioned for robotic knee arthroplasty and his comfort was confirmed. The surgery proceeded with minimal sedation so as not to obliterate pharyngeal reflexes and ensure the patient remained responsive to verbal stimuli. Most of the time, even with spinal anesthesia for lower limb arthroplasty, patients would be under deep sedation, but in this case, extra care was taken to avoid this to keep airway reflexes intact. Following an uneventful surgery, the patient was stable and comfortable in the post-anesthesia care unit (PACU). He was discharged home later in the day. Without gastric POCUS imaging with this patient, we would not have known about the significant residual gastric volume. 

## Discussion

The number of patients on GLP-1 RA has increased significantly in recent times as an effective advancement in diabetes management and widespread use for obesity. At the same time, anesthesiologists are concerned with perioperative management of these patients due to delayed gastric emptying resulting in residual gastric contents, increasing the risk of airway aspiration 1, 2, 3, 4.

All three patients we discussed had increased residual gastric contents or volume despite following guidelines to stop injectable GLP-1 RA for a week prior to surgery. Preoperative gastric POCUS exams revealed their increased risk for aspiration and enabled us to formulate or change the anesthetic plan for safer airway management. Being a minor elective procedure, case #1 was cancelled and was rescheduled for a later date. In case #2, we proceeded with an alternative, protective airway management option with endo tracheal tube to prevent airway aspiration especially with the surgery being performed in beach chair sitting position. In case #3, suitable regional anesthesia techniques that did not compromise airway reflexes were utilized to proceed with lower extremity surgery. The information from gastric POCUS enabled us to alter and personalize anesthetic management, depending on the urgency or nature of surgery and the final decision-making ability to provide safe anesthetic care.

Weekly injectable GLP-1 RA have long half-lives. The available recommendations 5 to stop GLP-1 RA for a week before surgery may not be adequate in all patients 6. It may also be difficult to stop these medications for longer than a week if a patient’s glycemic control depends on it. Preoperative gastric POCUS might be the only available tool to provide an objective assessment of gastric contents in these patients at higher risk. 

Another question or uncertainty is if the normal fasting recommendations are adequate for these patients who have delayed gastric emptying with GLP-1 RA. We do not have data if longer than normal preoperative fasting period or liquid diet for 24-hours preop (as recommended for colonoscopy) would benefit these patients. As the editorial by Jones et al. points out, we need to “proceed with caution” in patients taking GLP-1 RA 7.

The presence of solid or high-volume gastric fluid contents (gastric antral cross-sectional area more than 10 cm^2^) may warrant cancellation or rescheduling of a surgery. This raises the questions: Should these patients hold their GLP-1 RA for longer than a week for weekly injectable medications? And how long for oral daily medications? More studies are needed to have clear data on the holding period for these medications in the perioperative period as well as on the fasting periods.

We strongly recommend a preoperative gastric POCUS for all patients on GLP-1 RA, irrespective of timing of the last dose of the medication and fasting status. Gastric POCUS provides objective information that can help formulate a safe anesthetic plan.

## Disclosure Statement 

The authors report no conflict of interest related to this article. 

## Statement of Informed Consent

Informed consent was obtained from all participants to use medical information and ultrasound images/clips without revealing identity. 

## Supplementary Material

• Video S1 Gastric case 1

• Video S2 Gastric case 2

• Video S3 Gastric case 3
